# Sweet cherry flesh cells burst in non-random clusters along minor veins

**DOI:** 10.1007/s00425-022-03882-7

**Published:** 2022-04-07

**Authors:** Tobias Brinkmann, Felix Kuhnke, Eckhard Grimm, Moritz Knoche

**Affiliations:** 1 Abteilung Obstbau, Institut für Gartenbauliche Produktionssysteme, Leibniz Universität Hannover, Herrenhäuser Str. 2, 30419 Hannover, Germany; 2 Institut für Informationsverarbeitung, Leibniz Universität Hannover, Appelstr. 9a, 30167 Hannover, Germany

**Keywords:** Cracking, Osmotic potential, Phloem, *Prunus avium*, Solute potential, Water uptake

## Abstract

**Main conclusion:**

**Sweet cherry flesh cells burst when exposed to water but they do so in clusters indicating heterogeneity with respect to osmotic concentration, which depends on proximity to a minor vein.**

**Abstract:**

Water plays a key role in cracking in sweet cherry fruit. Magnetic resonance imaging has previously indicated preferential partitioning of water along veins. A more negative osmotic potential along veins seems the likely explanation. Here we establish if cell bursting in mature sweet cherry fruit is also associated with the veins. Cell bursting was identified by a novel light microscope technique involving exposure of a cut fruit surface to water or to sucrose solutions. Upon exposure to water there was no bursting of skin cells but for cells of the flesh (mesocarp) bursting increased with time. When the cut surface was exposed to sucrose solutions of decreasing osmotic concentrations (increasing water potentials) the incidence of cell bursting increased from hypertonic (no bursting), to isotonic, to hypotonic. Cell bursting in the outer mesocarp occurred primarily in the vicinity of minor veins that in the inner mesocarp was primarily between radial veins. The median distance between a minor vein and a bursting cell (mean diameter 0.129 mm) was about 0.318 mm that between a radial vein and a bursting cell was about 0.497 mm. In contrast, the distance between adjacent minor veins averaged 2.57 mm, that between adjacent radial veins averaged 0.83 mm. Cell bursting tends to occur in clusters. Mapping of cell bursting indicates (1) that a seemingly uniform population of mesocarp cells actually represents a heterogeneous population with regard to their cell osmotic potentials and (2) cell bursting afflicts clusters of neighbouring cells in the vicinities of minor veins.

**Supplementary Information:**

The online version contains supplementary material available at 10.1007/s00425-022-03882-7.

## Introduction

Excessive wetness is strongly implicated in the phenomenon of cracking in many fruit crop species. In sweet cherry, fruit cracking is of two sorts, macrocracks and microcracks. Macrocracks are plainly visible to the eye as splits in the skin that can extend deep into the flesh, sometimes exposing the pit. Badly cracked fruit are rapidly degraded by fungal rots. Microcracks are visible only under the microscope where they appear as tiny splits in the outer layer of the cuticle. Microcracks can extend into the subtending epidermal cell layer. Exposure of the strained skin of a sweet cherry to high humidity is sufficient to induce microcracking (Knoche and Peschel 2006). Microcracks impair the barrier functions of the cuticle allowing uncontrolled egress/ingress of water (Knoche et al. 2000) and ingress of pathogens (Borve et al. 2000).

Water uptake into the fruit is a physical process driven by a gradient of water potential between, say, an external drop of rainwater or dew (~ 0 MPa) and the cells of the epidermis (~ −1.4 MPa for the skin vs. −2.6 MPa for the mesocarp) (Beyer and Knoche 2002; Grimm and Knoche 2015). As flesh turgor is negligibly low in a mature sweet cherry relative to its osmotic potential, a fruit’s water potential is numerically about equal its osmotic potential (Knoche et al. 2014). Recent evidence shows the osmotic potential of the flesh of a sweet cherry is more negative than that of the overlying skin layer-the epidermis (one cell layer thick) and the hypodermis (2 or 3 cell layers thick) (Grimm and Knoche 2015). Because water diffuses from a region of less negative water potential to one of more negative water potential, the flesh is a strong diffusional sink for excess water entering the skin. A macrocrack is usually initiated by the bursting of individual cells in the outer flesh layer located just beneath the skin and often in line with a cuticular microcrack (Grimm et al. 2019). These flesh cells are also much larger, so more severely stressed (*T* = *P* $\times$ *r* where *T* is wall tension, *P* the excess pressure in the cell, and r the cell radius; Lustig and Bernstein 1985; Lang and Düring 1990) and thinner-walled, so are mechanically much less able to withstand high turgor, so they burst.

It may be speculated that the reason why some cells in the outer flesh (outer mesocarp) burst, while others do not, is because of heterogeneity among them in osmotic potential and thus turgor. For example, a cell having a higher than average carbohydrate content would have a more negative osmotic potential and thus develop a higher turgor and/or a higher strain. Also, a cell in the immediate vicinity of a vein may have a more negative osmotic potential than one further away, and so will take up water more readily, and so will develop a higher turgor and/or a higher strain, and so will suffer a higher likelihood of bursting (Simon 1977). Throughout fruit development and to maturity, sucrose and sorbitol are imported into the fruit via the phloem (Gao et al. 2003; Knoche et al. 2004; Brüggenwirth et al. 2016). Distribution of these moieties within the fruit occurs via the minor vein network located in the outer mesocarp (Grimm et al. 2017). There, sucrose and sorbitol are unloaded and transferred to the surrounding bundle-sheath cells. It is not clear whether symplastic or apoplastic unloading or a combination of both prevails in sweet cherry. Regardless of the unloading mechanism the further distribution occurs by non-vascular, cell-to-cell transport via the plasmodesmata. Support for increasing negative osmotic potentials (increasing osmotic concentrations) distally along the veins comes from MRI imaging which reveals preferential partitioning of water along the veins (Grimm et al. 2020).

Here, we quantify and analyse the spatial distribution of bursting cells in the outer and inner mesocarp of sweet cherry with special reference to its vasculature. We focus on the network of peripheral vascular bundles that we refer to as the network of ‘minor veins’ and that occurs as a reticulum in the outer mesocarp and on the lateral veins that we refer to as the ‘radial veins’ in the inner mesocarp (Fig. S1). The minor vein reticulum runs parallel to the fruit surface at a distance of a few mm below the fruit skin and serves as the distribution phloem in analogy to the collection phloem of minor veins in leaves. The radial veins in the inner mesocarp connect the major bundles, i.e., the two lateral and the median bundle, in the vicinity of the pit to the minor vein reticulum in the outer mesocarp (Grimm et al. 2017). The function of these radial veins of the inner mesocarp is distribution and transport to feed the minor vein reticulum in the outer mesocarp.

## Materials and methods

### Plant material

Mature ‘Fabiola’ and ‘Regina’ sweet cherry fruits (*Prunus avium* L.) were sampled from 14-year-old trees grafted on ‘Gisela 5’ (*Prunus cerasus* L. × *Prunus canescens* Bios) rootstocks in an experimental orchard of the Leibniz University Hannover at Herrenhausen (52°23' N; 9°42' E). Trees were trained to slender spindle trees and cultivated according to current regulations for integrated fruit production. Individual visually perfect fruits were sampled at commercial maturity based on color and size 1–2 h before the beginning of an experiment.

### General experimental procedure

Radial or tangential cuts were made using a sharp razor blade. The cut surface of the fruit was carefully blotted dry using a soft paper tissue. The fruit segments were then mounted, cut surface uppermost and horizontal, in a petri dish using a drop of cyanoacrylate adhesive (Loctite 406; Henkel, Düsseldorf, Deutschland). The whole was then positioned under a binocular microscope (MZ 10 F; Leica, Wetzlar, Deutschland) equipped with a digital camera (DP 71; Olympus, Hamburg, Deutschland). The treatment solution (see below) was then placed on the cut surface taking care to exclude air bubbles. The cut surface was then viewed under incident light and photographed repeatedly at defined intervals over defined periods of time. The calibrated images were later processed by image analysis.

Multiple photographs were assembled to create a time-lapse video (VLC media player 3.0.11; VideoLAN, Paris, France). A burst cell was identified in the video based on the following criteria: a transient change of reflectance and/or a change in color and/or a sudden decrease in cell volume. Occasionally, bursting was also associated with a transient refractive blurring as the cell content leaked into the treatment solution. The location of each burst cell was recorded on the first (still) image (i.e., the one taken at time zero) using the open-source image editor GIMP 2.8 (www.gimp.org, retrieved on 11/21/2015).

### Experiments

Using the above procedure, the following experiments were conducted.

First, the time course of cell bursting following exposure of the cut surface to deionised water was established in ‘Fabiola’ and ‘Regina’ sweet cherries. Radial cuts parallel to the longitudinal axis of the fruit were made in the cheek region. The segments were mounted and the entire mesocarp viewed as described above. Deionized water was applied to the cut surface. Short-term time courses were conducted for up to 120 s with each cultivar (*n* = 4). In addition, in ‘Fabiola’, a long-term time course of up to 600 s was established (*n* = 3).

Second, the effect of the osmotic potential of the treatment solution on cell bursting was quantified. Radial sections were prepared and viewed as described above. The cut surface of a ‘Regina’ sweet cherry was then exposed for 120 s to one of a number of sucrose solutions of different concentration: 0, 600, 800, 1000, 1200 and 1400 mM. The osmotic potentials of the treatment solutions were determined by water vapor pressure osmometry (Vapro 5600; Wescor, Logan, UT, USA) as 0.0, −1.8, −2.6, −3.5 −4.6 and −6.0 MPa, respectively. The water potential of the expressed juice of the ‘Regina’ fruit of this batch was −3.9 ± 0.1 MPa (*n* = 20). The experiment was conducted twice. In the first run, a randomised ‘single-measure’ design was used where a different fruit was used for each treatment solution concentration. In the second run, a ‘repeated-measures’ design was used, where the osmotic potential of the treatment solution was increased (concentration was decreased) stepwise from hypertonic (−6.0 MPa) to water (0.0 MPa). The number of burst cells was determined 120 s after the beginning of exposure to each new treatment solution. Both experiments were carried out with three replicates.

Third, frequency distributions of the distances between burst cells and the tangential reticulum of minor veins in the outer mesocarp and the radial veins in the inner mesocarp were established in ‘Regina’ sweet cherry. Tangential cuts were made using a sharp blade aligned parallel to the fruit’s longitudinal axis and parallel to the fruit surface, so as to remove a ‘spherical cap’ of tissue from the fruit’s cheek. The caps were discarded. The cut surface of the fruit was carefully blotted dry using a soft paper tissue. Provided that the sections were sufficiently thin, the tissue seen in the center of this roughly circular cut surface were of the outer mesocarp revealing the reticulum of minor veins. For the radial veins in the inner mesocarp, a single tangential cut parallel to the fruit surface was made closer to the pit. Following removal of the tissue, cross-sections of radial veins were exposed on the cut surface. The cut surface was viewed as described above. The closest neighbouring vein to the bursting cell was identified and its distance to the cell determined and the frequency distribution established. The mean half-distances between minor veins and the mean half-distances between radial veins served for comparison. The latter were determined for those minor veins of a loop of the reticulum that were within the focal plane of the image. Here, two orthogonal distances per loop of the reticulum were determined (Fig. S2a). In the inner mesocarp, the distances between radial veins were determined (Fig. S2b).

Fourth, a possible clustering of cell bursting was investigated in radial and tangential sections through the outer and inner mesocarp of ‘Fabiola’ and ‘Regina’ sweet cherry. Working in the 2D plane of the cut flesh surface, frequency distributions of the distances between pairs of adjacent burst cells were determined. To do this, the position of each burst cell was first expressed in terms of a pair of *x–y* coordinates. Next, for each burst cell, the distances to all other burst cells were calculated. The distance to the nearest burst cell was computed and the frequency distribution of the distances so obtained was calculated. The geometrical mean cell diameter was determined for each cell as the square root of the product of each pair of orthogonal diameters.

### Data analyses and presentation

Data are presented in the figures as means and standard errors of means.

## Results

**Fig. 1:**
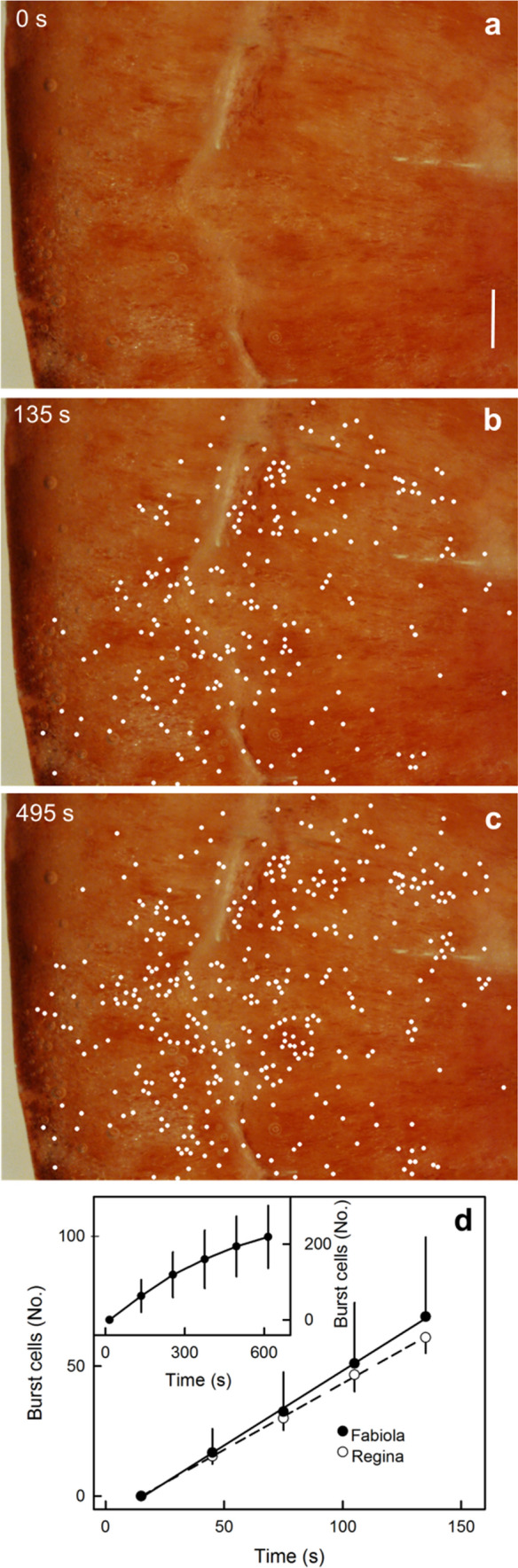
**a**–**c** Light micrographs taken from above the cut surface of a ripe ‘Fabiola’ sweet cherry fruit exposed to deionised water. White dots indicate burst cells detected by inspection of a temporal sequential of light micrographs. Bar 1 = mm. **d** Short period of exposure to deionised water. Inset: longer period of exposure to deionised water. Cell bursting was detected by observing differences between sequential light micrographs. Data represent means ± SE of three (main graph) and four replicates (inset). For details see text

When the cut surface of a mature sweet cherry was exposed to water, the number of burst cells in the outer mesocarp increased steadily with time (Fig. 1a–c). There was very little evidence of bursting of the cells in the skin. The number of burst cells in the outer mesocarp increased linearly with time up to 120 s in both ‘Fabiola’ and ‘Regina’ sweet cherry (Fig. 1d). The rate of bursting decreased slightly if the incubation period was extended to 600 s, with the cumulative number of burst cells starting to level off (Fig. 1d, inset).

**Fig. 2:**
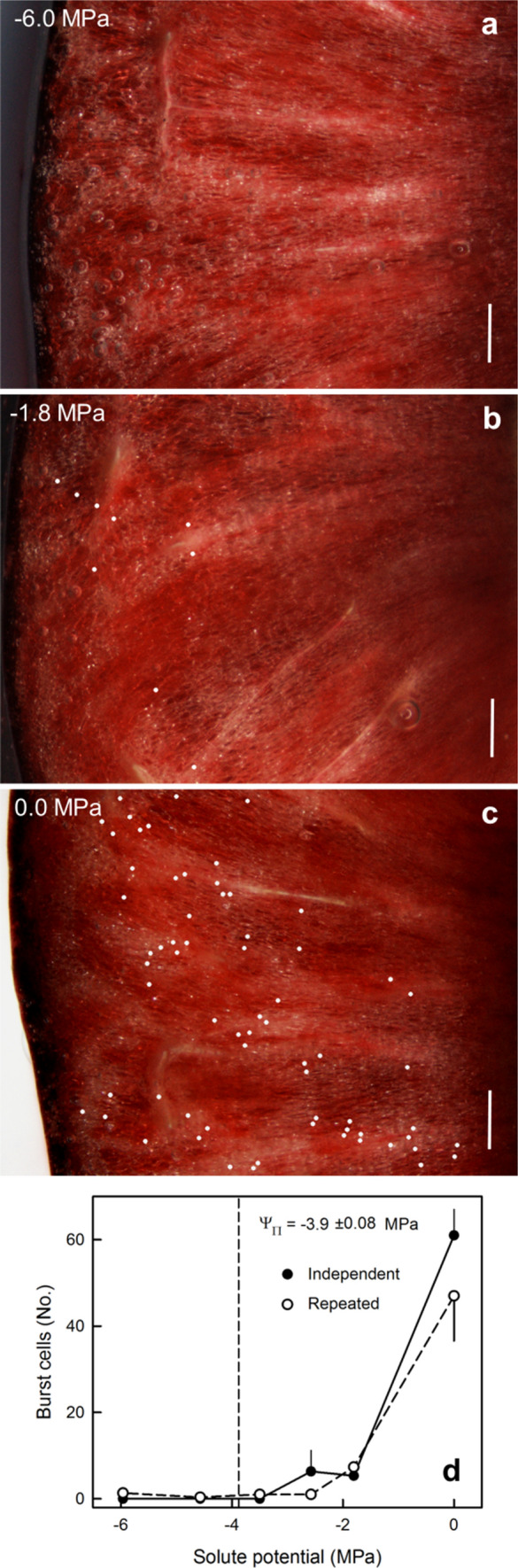
**a**–**c** Light micrographs taken from above the cut surface of ripe ‘Regina’ sweet cherry fruits exposed to sucrose solutions of decreasing osmotic concentration (less negative osmotic potential). Bar = 1 mm. **d** Effect of osmotic potential on the incidence of cell bursting. Vertical dashed lines indicate the osmotic potential of expressed sweet cherry juice. Cell bursting was detected by light microscopy. Cell bursting was recorded for experimental designs employing either ‘single-measures’ or ‘repeated-measures’. Data represent means ± SE of three replicates. For details see text

Exposure to sucrose solutions of decreasing osmotic concentration (less negative osmotic potential) resulted in increasing incidences of cell bursting (Fig. 2a–c), regardless of whether the experiment was conducted using the ‘single-measures’ or the ‘repeated-measures’ design (Fig. 2d). There was no bursting of cells when the cut surface was exposed to a hypertonic sucrose solution.

**Fig. 3:**
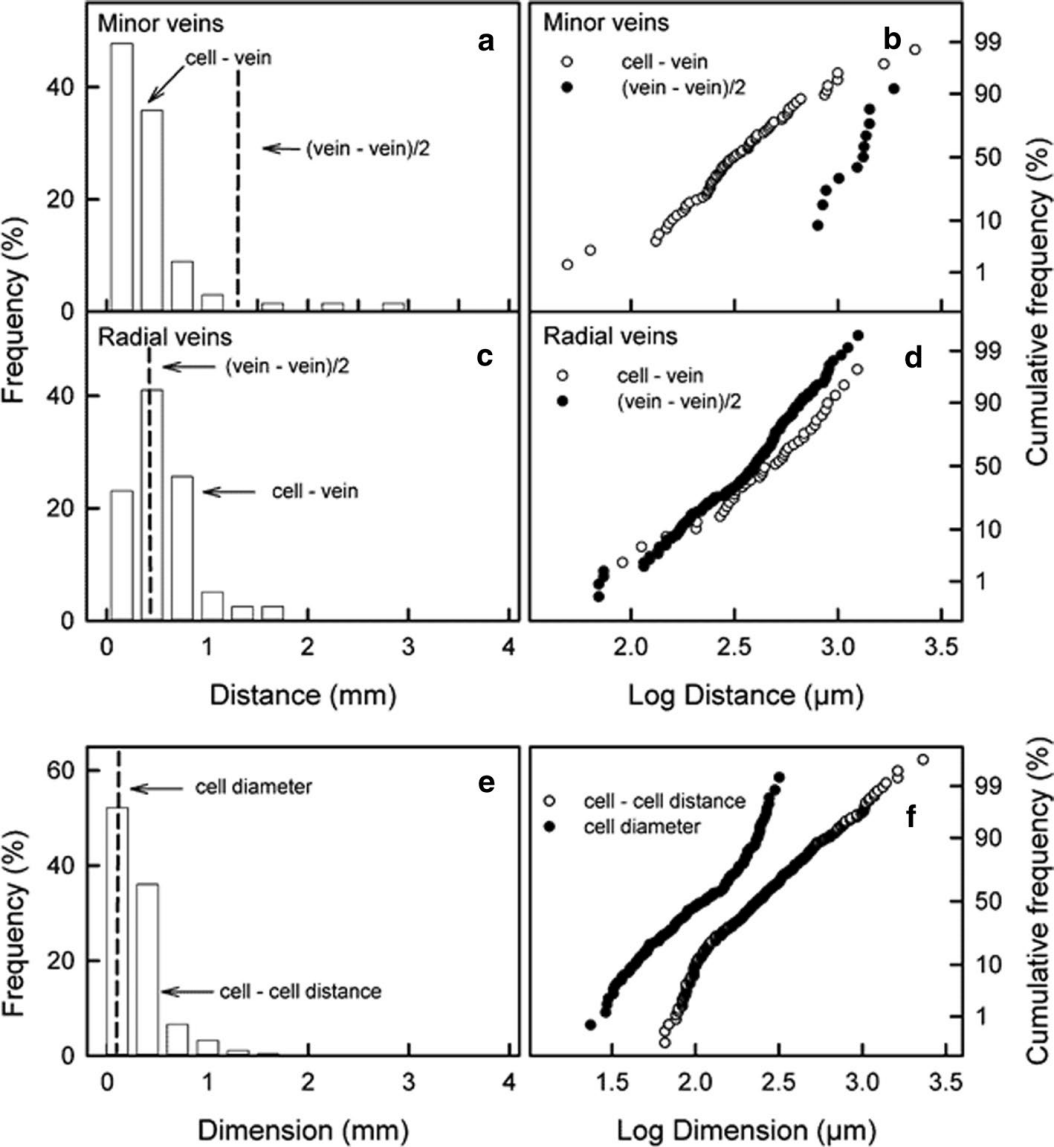
**a**–**d** Frequency distributions of the distances between (**a, b**) burst cells and minor veins of the outer mesocarp or (**c, d**) burst cells and the radial veins of the inner mesocarp of ripe sweet cherries. The vertical dashed lines in **a** and **c** indicate the mean half-distance [‘(vein–vein)/2’] between two minor veins (**a**) and between two radial veins (**c**). **b, d** Same data as in **a** and **c** but redrawn as cumulative frequency distributions of log transformed distances. Note that the *y*-axis is plotted on a probability scale. Linearity indicates distances are log normal distributed. **e** Frequency distribution of minimum distances between burst cells (‘cell–cell distance’) in the outer mesocarp of ripe sweet cherries. The vertical dashed line in **e** indicates mean diameter of the outer mesocarp cells (‘cell diameter’) (*n* = 162). **f** Cumulative frequency distributions of log transformed minimum distances between burst mesocarp cells (○) and of mesocarp cell diameters (●)

Cell bursting occurred primarily in the immediate vicinity of the minor veins in the outer mesocarp and between the radial veins in the inner mesocarp. The median distance between a minor vein and a burst parenchyma cell was 0.318 mm, that between a radial vein and a burst cell was 0.497 mm (Fig. 3a, c). The distances between minor or radial veins and burst cells followed log normal distributions as indexed by the linearity of the cumulative normal probability plots (Fig. 3b, d). The mean half-distance between minor veins of the outer mesocarp averaged 1.272 ± 0.125 mm and was about four-times larger than the median distance between a burst cell and an adjacent minor vein. The spacing of radial veins in the inner mesocarp was closer. Here, the mean half-distance was only 0.414 ± 0.014 mm and approximately equal to the median distance between neighbouring bursting events (Fig. 3d).

The frequency distributions of distances between bursting mesocarp cells indicate ‘spatial clustering’ of bursting events. Cell bursting occurred most often within 0.300 mm of a burst cell. This distance corresponds approximately to the diameters of two outer mesocarp cells (mean diam. 0.111 ± 0.04 mm) (Fig. 3e). The distances between bursting outer mesocarp cells also follow a log normal distribution (Fig. 3f).

## Discussion

The bursting of a mesocarp cell results from water uptake by the cell, driven by a gradient in water potential between the solution applied to the fruit’s cut surface and that of the contents of the cell (lying just below the cut surface). The inner and outer mesocarp cells are large, have thin cell walls and their symplast osmotic potentials are strongly negative. The skin cells (epidermis and hypodermis) differ from cells of the inner and outer mesocarp in that they are smaller, have thicker walls and less negative osmotic potentials (less negative by about −1.1 MPa) (Grimm and Knoche 2015; Brüggenwirth and Knoche 2016). When the limit of extensibility of a cell wall is exceeded (i.e., the breaking strain) it will burst. The cell contents will then erupt into the apoplast and thus mix with the treatment solution flooding the cut surface of the tissue. The cell bursting was clearly visible under the light microscope.

### Factors affecting cell bursting

The cumulative number of burst mesocarp cells increased with time. In the short term, this increase was linear (a constant rate of bursting). Over a longer term, however, we noted that the frequency of cell bursting started to decrease. This could have been for two reasons. First, for longer exposure times the bursting events became progressively more difficult to identify and thus to quantify due (i) to swelling of the cells and cell walls which caused slight shifts in the microscope image and, hence, changes in the recorded *x*–*y* coordinates of a burst cell and (ii) an increasing lack of structure in the images as more and more juice accumulated on the cut surface (Fig. S3a, b). A second factor could have been an increasing accumulation in the treatment solution of leaked cell contents. This would have affected its (i) osmotic concentration (making it more concentrated) and (ii) an increased presence of malate already established as being responsible for cell-wall swelling and weakening (Winkler et al. 2015).

The frequency of bursting was also affected by the osmotic potential of the solution applied to the cut surface. There was no cell bursting, when solutions were applied that were hypertonic to the expressed juice of the fruit (−3.9 MPa, see above). Bursting occurred only when hypotonic solutions were applied. This behaviour was expected. The turgor of a mesocarp cell is negligibly low (compared with, say, a healthy mesophyll cell) and hence the water potential of a mesocarp cell is essentially equal to the osmotic potential of its symplast (Knoche et al. 2014; Schumann et al. 2014). Water uptake from a hypotonic solution occurs, as only then will there be a gradient in water potential towards the cell. Our observation here that some anthocyanin leakage may also occur into an isotonic solution was earlier reported by Winkler et al. (2015).

The absence of bursting of the skin cells is due in part to their less negative osmotic potential (compared to the mesocarp cells; Grimm and Knoche 2015) and also to their greater strength (much smaller, much thicker cell walls) (Brüggenwirth and Knoche 2016). This observation indicates significant heterogeneity in the osmotic potentials of the component cells among the fruit’s major tissues. However, our focus is on the cell:cell heterogeneity within the mesocarp tissue (Grimm and Knoche 2015; Grimm et al. 2019).

### Evidence for increased incidence of cell bursting along minor veins

Our results evidence an increased incidence of cell bursting along the minor veins embedded in the outer mesocarp. In the inner mesocarp, the highest incidence of cell bursting occurred about halfway between adjacent radial veins.

#### Outer mesocarp

For the reticulum of minor veins in the outer mesocarp, the incidence of cell bursting decreased as the distance from a minor vein increased. Bursting was maximal close to (0 and 0.300 mm) a minor vein. The half-diameter of a ‘loop’ of the minor vein reticulum was 1.272 ± 0.125 mm. This clearly indicates a clustering of cell bursting immediately adjacent to a minor vein. Assuming the morphology of the mesocarp cells is uniform, the most likely explanation for our observation of clustering is an increased driving force for water uptake. A larger driving force resulting from a steeper water potential gradient between the symplast of the cell (just before bursting) and of the treatment solution applied to the cut surface. The reticulum of minor veins represents the endpoint of the phloem pathway entering the fruit from the tree. The sieve tubes here function as the unloading network of the phloem (Ma et al. 2019). In fruit, sieve tube unloading may occur directly into the symplast of the adjacent cells via plasmodesmata and/or first into the apoplast (cell wall free space) then followed by uptake into the symplast of these adjacent cells (Ma et al. 2019; Falchi et al. 2020). The compartmentation and metabolism of carbohydrates is known to be somewhat variable among the stone fruit species, it is likely species-specific and it has not yet been studied in sweet cherry (Walker et al. 2020). In sour cherry (*Prunus cerasus*), sorbitol carriers have been identified that make apoplastic unloading likely (Gao et al. 2003). In Japanese plum (*Prunus salicina*) unloading is apoplastic and symplastic (Grappadelli et al. 2019). Regardless of the unloading mechanism in our case (viz symplastic or apoplastic), the most negative osmotic potentials (the highest osmotic concentrations) are expected in cells close to the minor veins of the outer mesocarp where sieve tube unloading is occurring.

#### Inner mesocarp

In contrast, for the inner mesocarp, cell bursting was maximal between two adjacent radial veins. The maximum incidence of bursting occurred between 0.300 and 0.600 mm which corresponds to a mean half-distance between adjacent radial veins of 0.414 ± 0.014 mm. Consequently, any bursting event in the inner mesocarp recorded beyond the mean half-distance between radial veins must be considered artefactual and thus should be interpreted with caution. The reason for an increase in the incidence of cell bursting at some distance from the radial veins is not known. It is speculated that apoplastic solutes may affect the water potential gradient and so determine the water uptake of the inner mesocarp cells. Such elevated apoplastic solutes may result either from leakage from the phloem or apoplastic unloading of the phloem (Cabrita et al. 2013). Any apoplastic solutes would thus combine with the treatment solution applied to the cut surface. That apoplastic solutes occur naturally in maturing sweet cherries is not unlikely considering the absence of significant turgor of the mesocarp cells (Wada et al. 2008, 2009; Knoche et al. 2014).

Additionally, if solute transport away from mesocarp cells located between radial veins was sluggish these would develop more negative water potentials and, hence, suffer an increased likelihood of cracking.

### Evidence for clustering of cell bursting

The bursting of mesocarp cells occurs in clusters. The bursting of an individual mesocarp cell makes it more likely that cells in its vicinity will soon follow (i.e., within a distance of about two cell diameters). There are three explanations that can be offered. First, based on the zipper model (Knoche and Winkler 2017), a bursting cell releases malic acid into the apoplast (Winkler et al. 2015). Malic acid increases the permeability of the plasma membranes of adjacent cells, thereby causing further bursting and leakage (Winkler et al. 2015). In addition, malic acid weakens cell-to-cell adhesion by extracting Ca from the cell wall (Brüggenwirth and Knoche 2017). Furthermore, the loss of turgor as a consequence of cell bursting results in a further swelling of cell walls and a further weakening of cell-to-cell adhesion (Schumann and Knoche 2020). This sequence of events results in a chain reaction that causes a crack to ‘run’ and the tissue to ‘unzip’ (Schumann et al. 2019). Second, adjacent cells will have the same proximity to a vein. Thus, the osmotic gradients driving water uptake are expected to be similar for adjacent cells. Third, a pair of adjacent cells will likely be daughter cells of an earlier cell division so may share some filial connection in their physiologies.

## Conclusion

The mapping of bursting mesocarp cells near the cut surface of a mature sweet cherry fruit provides a new tool for identifying heterogeneity in a seemingly homogeneous tissue. Using this method, we observed a clustering of cell bursting events along the minor veins in the outer mesocarp. We conclude that the higher frequency of cell bursting in the vicinity of a minor vein is accounted for by these cells having osmotic potentials that are more negative than those of most other outer mesocarp cells. This, in turn, is likely caused by an imbalance between the rate of solute unloading from the sieve tubes and the rate of cell-to-cell solute transport via plasmodesmata. Apparently, the rate of unloading of the sieve tubes exceeds the rate of cell-to-cell transport into the mesocarp cells. This conclusion has important implications for fruit cracking. It could provide an explanation for the high variability of fruit cracking susceptibility from tree to tree, from year to year and from site to site-even within the same genotype. Cell-to-cell heterogeneity in solute concentration is likely affected by multiple environmental variables (including light and temperature) and their interactions with most physiological process (including with photosynthesis and phloem transport). Also likely involved are multiple orchard factors (including management of crop load, canopy and irrigation).

### *Author contributions statement*

EG and MK conceived and designed the experiments. EG performed the experiments. TB, FK conducted the measurements. TB and EG analysed the data. EG and MK wrote the manuscript. All authors read and approved the manuscript.

## Supplementary Information

Below is the link to the electronic supplementary material.

Supplementary file1 (DOCX 3018 KB) Fig. S1 Sketch of sweet cherry fruit illustrating the different cuts made (a, b) and the veination in a sweet cherry fruit (c, d). a Tangential cuts made in the outer (dotted line) and the inner mesocarp (dashed line) parallel to the fruit surface. b Radial cut along the longitudinal axis of the fruit. c, d Cross section of a sweet cherry fruit revealing veination. c Overview. d Detailed view revealing the peripheral minor vein (‘mv’) reticulum in the outer mesocarp underneath the skin and the lateral radial veins (‘rv’) in the inner mesocarp. For further details see text and Grimm et al. (2017). Fig. S2 Light micrographs taken from above the cut surface of the outer mesocarp of ripe ‘Regina’ sweet cherry fruit (a) showing a reticulum (network) of minor veins. b Same as a but above the inner mesocarp. Bar = 1 mm. White lines in a indicate distances between minor veins in the peripheral reticulum. White dots in b indicate radial veins. For both micrographs a tangential cut parallel to the fruit surface was made. The section in a was produced from a cut close and parallel to the fruit surface in the outer mesocarp (dotted line in Fig. S1a), that in b from a parallel cut in the inner mesocarp (dashed line in Fig. S1a). Fig. S3 Light micrographs taken from above the cut surface of the outer mesocarp of ripe ‘Fabiola’ sweet cherry fruit in contact with deionised water for 0 min and 10 min. Bar = 1 mm

## Data Availability

The datasets generated during and/or analysed during the current study are available from the corresponding author on reasonable request.
