# Supplementary figures and images for: Sweet cherry flesh cells burst in non-random clusters along minor veins
Source: Planta. 2022 Apr 7;255(5):100. doi: 10.1007/s00425-022-03882-7 (PMC8990966; doi:10.1007/s00425-022-03882-7)

**Supplemental Figure**


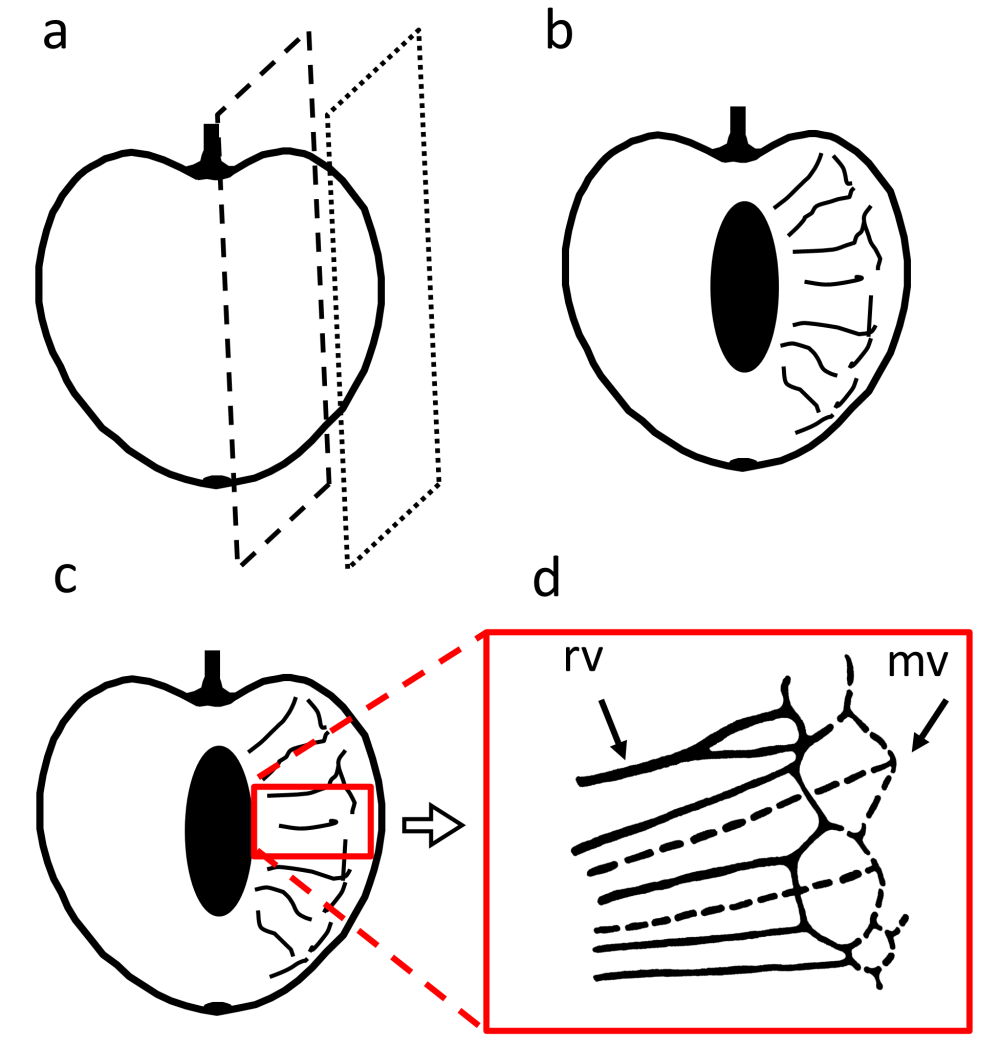


C

A

**Figure S1.**


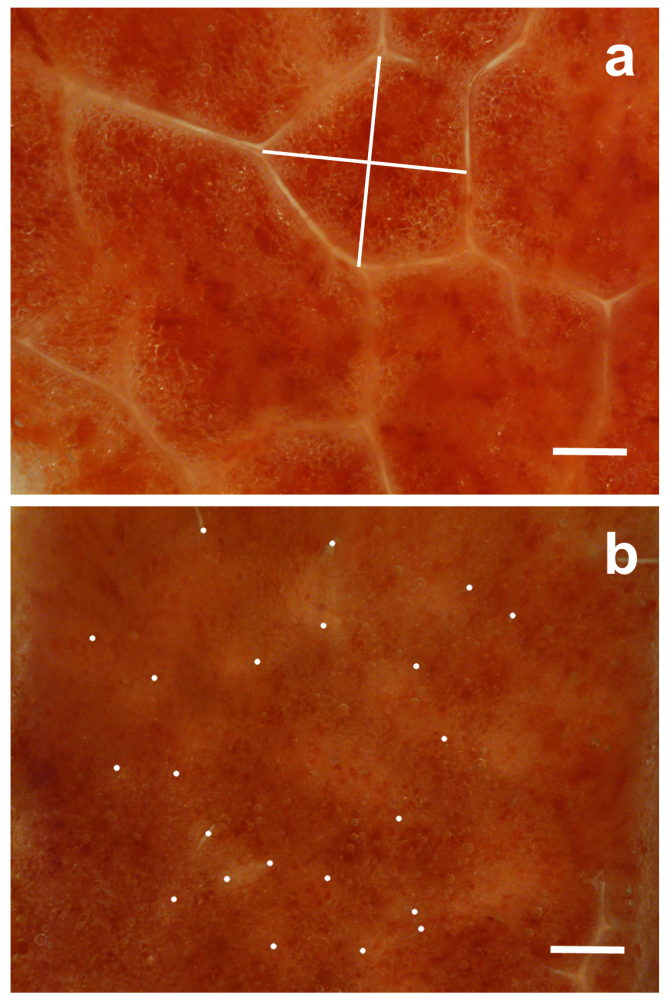


**Figure S2.**

**
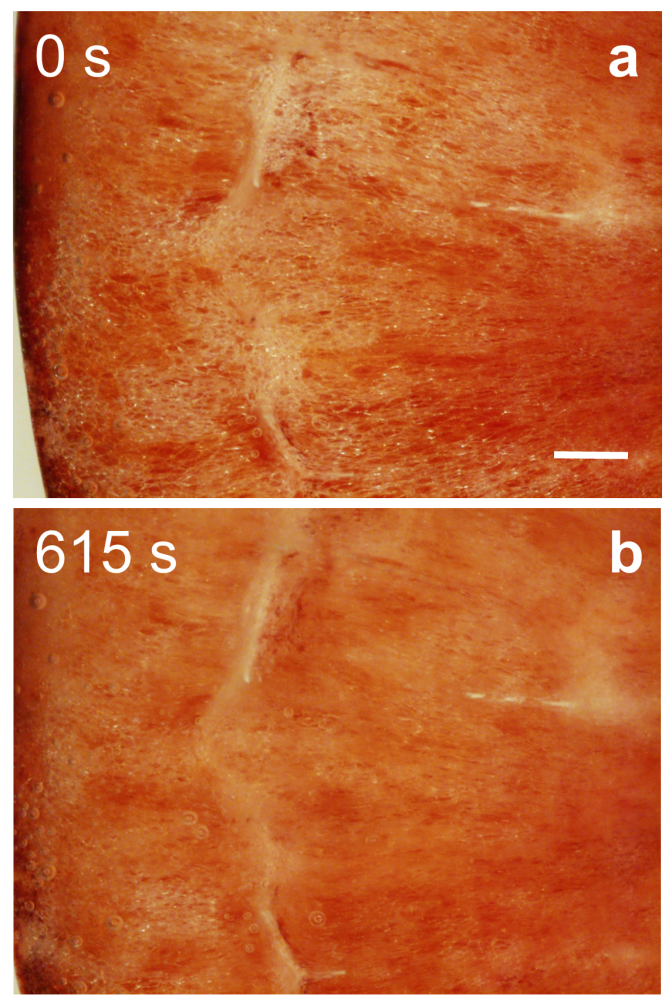
**

**Figure S3.**

Supplement: Supplementary file 1 — Supplementary file1 (DOCX 3018 KB) Fig. S1 Sketch of sweet cherry fruit illustrating the different cuts made (a, b) and the veination in a sweet cherry fruit (c, d). a Tangential cuts made in the outer (dotted line) and the inner mesocarp (dashed line) parallel to the fruit surface. b Radial cut along the longitudinal axis of the fruit. c, d Cross section of a sweet cherry fruit revealing veination. c Overview. d Detailed view revealing the peripheral minor vein (‘mv’) reticulum in the outer mesocarp underneath the skin and the lateral radial veins (‘rv’) in the inner mesocarp. For further details see text and Grimm et al. (2017). Fig. S2 Light micrographs taken from above the cut surface of the outer mesocarp of ripe ‘Regina’ sweet cherry fruit (a) showing a reticulum (network) of minor veins. b Same as a but above the inner mesocarp. Bar = 1 mm. White lines in a indicate distances between minor veins in the peripheral reticulum. White dots in b indicate radial veins. For both micrographs a tangential cut parallel to the fruit surface was made. The section in a was produced from a cut close and parallel to the fruit surface in the outer mesocarp (dotted line in Fig. S1a), that in b from a parallel cut in the inner mesocarp (dashed line in Fig. S1a). Fig. S3 Light micrographs taken from above the cut surface of the outer mesocarp of ripe ‘Fabiola’ sweet cherry fruit in contact with deionised water for 0 min and 10 min. Bar = 1 mm [file 425_2022_3882_MOESM1_ESM.docx]
